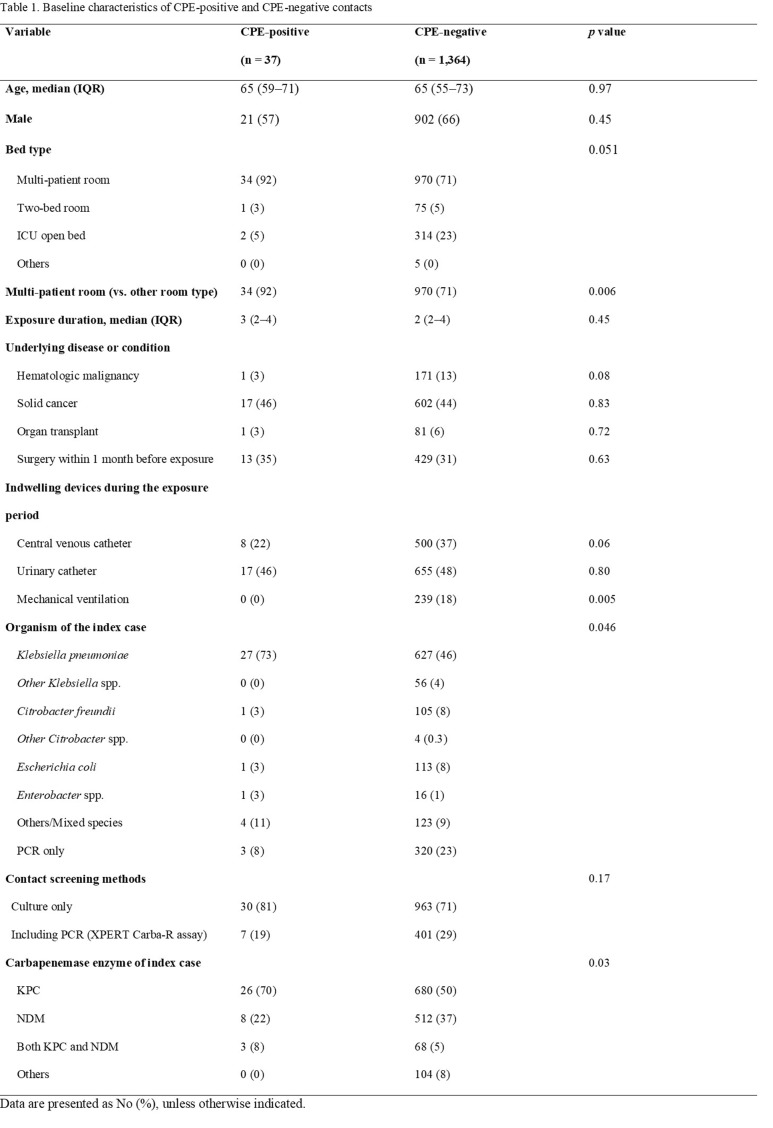# 177 SMART-BCx: System-wide Management Algorithm to Reduce Testing of Blood Cultures

**DOI:** 10.1017/ash.2026.10724

**Published:** 2026-06-23

**Authors:** Subeen Moon, Miseo Kim, Soyeon Park, Jeeyoon Kim, Mi-Na Kim, Heungsup Sung, Jiwon Jung, Yong Pil Chong

**Affiliations:** 1 Office for Infection Control, Asan Medical Center, Seoul, Korea; 2 Department of Laboratory Medicine University of Ulsan College of Medicine and Asan Medical Center

## Abstract

**Background:** Carbapenemase-producing Enterobacterales (CPE) spread silently and offer limited treatment options, making contact screening essential. We evaluated the real-world implementation of contact screening, focusing on screening timing (at exposure vs. at readmission) and positivity rates by room type and carbapenemase enzyme. **Methods:** This retrospective observational study was conducted at a 2,700-bed tertiary care hospital and included patients identified as contacts of CPE index cases with exposure between 1 January and 31 May 2024. Contacts were defined as patients who shared a room or occupied open beds in the same intensive care unit (ICU) with an index case during the period from 4 days before the index patient’s positive specimen collection until the reporting of results. Screening was performed with stool or rectal swabs either at the time of exposure or upon readmission within 6 months. CPE acquisition was defined as detection of isolates harbouring the same carbapenemase enzyme as the corresponding index case. Acquisition rates were compared by screening timing, room type, and carbapenemase enzyme. Samples were processed using culture or the Xpert Carba-R assay. **Result:** Among 2,003 contacts linked to 336 index patients, 1,401 (70%) underwent screening, of whom 37 (2.6%) tested positive. Immediate screening identified 30/1,184 (2.5%) positives, while readmission screening detected 7/217 (3.2%); two additional acquisitions were identified from clinical specimens among unscreened readmitted contacts. Acquisition was more frequent after exposure to KPC-producing organisms than NDM-producing organisms (3.7% [26/706] vs. 1.5% [8/520]; p=0.02) and higher in multi-patient rooms than in two-bed or open-bed ICU settings combined (3.4% [34/1004] vs 0.8% [3/397]; p=0.006). **Conclusion:** CPE acquisition was more likely following exposure to KPC-producing organisms and in multi-patient rooms. Systematic readmission screening identified additional carriers who would otherwise have been missed. These findings support tailored infection-prevention strategies that consider carbapenemase enzyme and the physical care environment, particularly in healthcare systems where multi-patient rooms are common.

**Figure:**
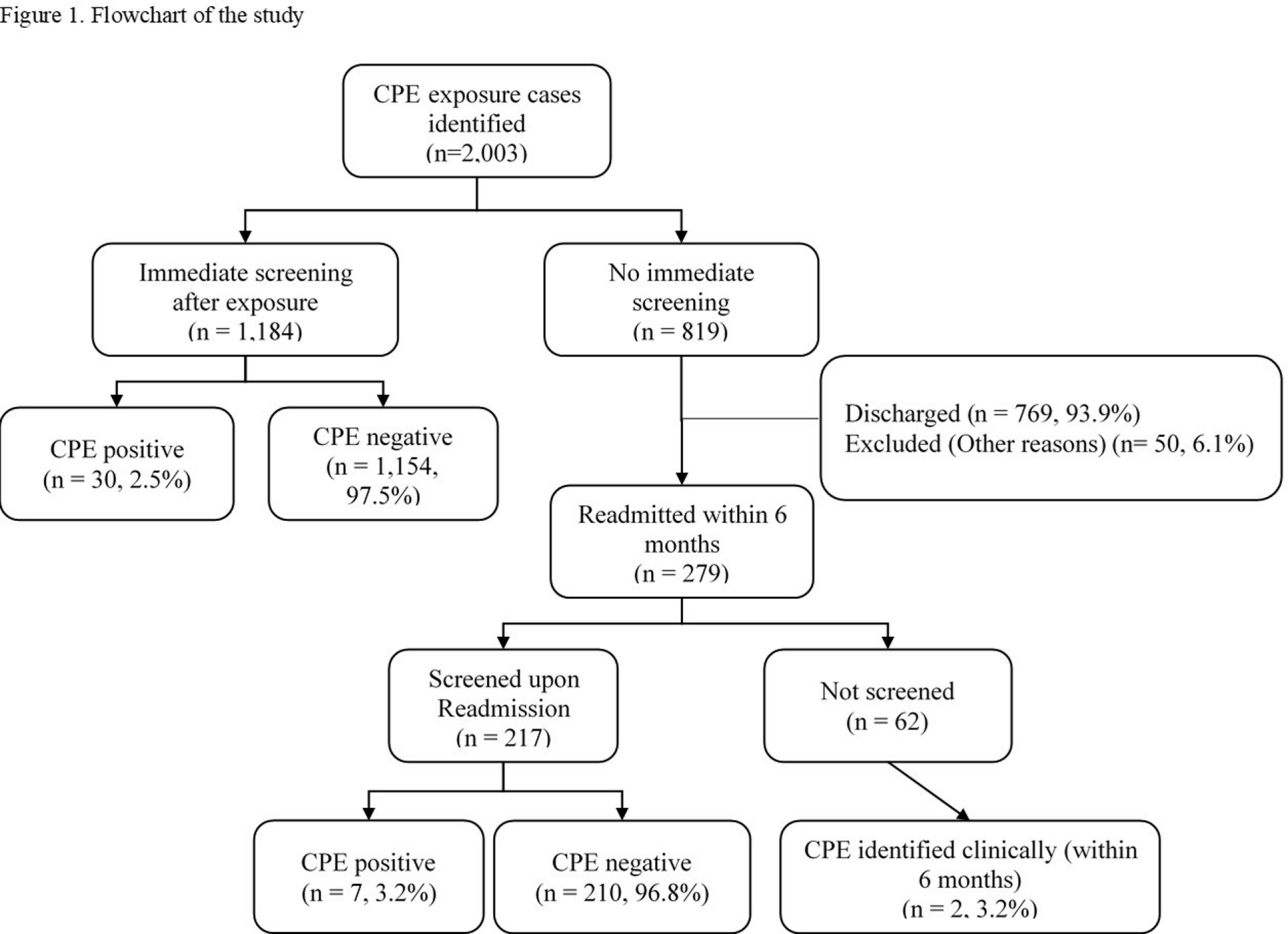

**Figure:**